# How Human Brucellosis Incidence in Urban Kampala Can Be Reduced Most Efficiently? A Stochastic Risk Assessment of Informally-Marketed Milk

**DOI:** 10.1371/journal.pone.0014188

**Published:** 2010-12-01

**Authors:** Kohei Makita, Eric M. Fèvre, Charles Waiswa, Mark C. Eisler, Susan C. Welburn

**Affiliations:** 1 Centre for Infectious Diseases, Division of Pathway Medicine, School of Biomedical Sciences, College of Medicine and Veterinary Medicine, The University of Edinburgh, Edinburgh, United Kingdom; 2 Ashworth Laboratories, School of Biological Sciences, The University of Edinburgh, Edinburgh, United Kingdom; 3 Faculty of Veterinary Medicine, Makerere University, Kampala, Uganda; Kenya Medical Research Institute, Kenya

## Abstract

**Background:**

In Kampala, Uganda, studies have shown a significant incidence of human brucellosis. A stochastic risk assessment involving two field surveys (cattle farms and milk shops) and a medical record survey was conducted to assess the risk of human brucellosis infection through consumption of informally marketed raw milk potentially infected with *Brucella abortus* in Kampala and to identify the best control options.

**Methodology/Principal Findings:**

In the cattle farm survey, sera of 425 cows in 177 herds in the Kampala economic zone were sampled and tested for brucellosis using a competitive enzyme-linked immunosorbent assay (CELISA). Farmers were interviewed for dairy information. In the milk shop surveys, 135 milk sellers in the urban areas were interviewed and 117 milk samples were collected and tested using an indirect enzyme-linked immunosorbent assay (IELISA). A medical record survey was conducted in Mulago National Referral Hospital for serological test results. A risk model was developed synthesizing data from these three surveys. Possible control options were prepared based on the model and the reduction of risk was simulated for each scenario. Overall, 12.6% (6.8–18.9: 90%CI) of informally marketed milk in urban Kampala was contaminated with *B.abortus* at purchase and the annual incidence rate was estimated to be 5.8 (90% CI: 5.3–6.2) per 10,000 people. The best control option would be the construction of a milk boiling centre either in Mbarara, the largest source of milk, or in peri-urban Kampala and to ensure that milk traders always sell milk to the boiling centre; 90% success in enforcing these two options would reduce risk by 47.4% (21.6–70.1: 90%CI) and 82.0% (71.0–89.0: 90%CI), respectively.

**Conclusion/Significance:**

This study quantifies the risk of human brucellosis infection through informally marketed milk and estimates the incidence rate in Kampala for the first time; risk-based mitigation strategies are outlined to assist in developing policy.

## Introduction

Brucellosis is one of the world's most widespread zoonoses [1]. In Kampala, Uganda, studies have shown a significant incidence of human brucellosis, and poor correlation between the spatial distribution of human cases (which tend to be in urban areas) and the cattle reservoir (which tend to be in peri-urban and rural areas) that is suggestive of brucellosis infection occurring through dairy value chains [2].

Brucellosis in cattle is endemic throughout Uganda. In 1972, the cattle prevalence was 16.3% in Ankole (southwest), 12.4% in Karamoja (northeast), 19.7% in West Nile (northwest) and 18.7% in Tororo (southeast) using the complement fixation test [3]. A few years later, using serum agglutination tests, the cattle prevalence was 19.7% in East Ankole (western), 23.3% in East Acholi (north), 9.0% in East Lango (north), 16.2% in Bulemezi (central) and 1.0% in Entebbe (central) [4]. Southern Uganda (Rukungiri District) is also endemic, with a herd level prevalence 7.5% and animal level prevalence of 3% having been reported [5]. This endemic situation has not altered until recently; in the central and southern parts of the country, a high prevalence of brucellosis at the herd level (56.3%) and at the animal level (5.0%) has been reported using both the Rose Bengal Test (RBT) and the serum agglutination test [6]. More recently, in urban and peri-urban areas of Kampala, 42% of cattle serum samples were shown to be positive using the slow serum tube agglutination test [7]. In the milk basin of Kampala and in the largest dairy production area in Uganda, Mbarara, a high cattle herd prevalence of brucellosis (55.6%: 95% CI 50.0–61.2) has been reported [8]. In effect milk from any part of Uganda may contain *Brucella* unless it is properly boiled. In Uganda, 92% of marketed milk passes through informal channels as unpasteurized milk or milk products [9] and Kampala, the capital, is not an exception.

There are 4 zoonotic ‘species’ (genetically regarded as the variants of *Brucella melitensis*) in genus *Brucella*. *B. abortus* is normally associated with cattle, *B. melitensis* with sheep and goats, *B. suis* with swine and *B. canis* with dogs [10]. The present study assesses the risk for brucellosis from dairy milk and thus *B. abortus* is the most relevant. Brucellosis in humans is characterised by an acute or sub-acute febrile illness usually marked by an intermittent or remittent fever accompanied by malaise, anorexia and prostration, and which, in the absence of specific treatment, may persist for weeks or months. Typically, few clinical signs are apparent but enlargement of the liver, spleen and/or lymph nodes may occur [10]. The disease can be treated using antibiotics [11]. Generally, human infection occurs through consumption of poorly prepared meat and dairy products in the form of milk, cheese and butter, although certain occupations such as veterinarians, butchers, abattoir workers, meat inspectors, farmers and those working in meat packing and dairy processing industries are known to be at a greater risk [12].

Control of brucellosis in cattle in developing countries in an endemic situation is not straight forward as mass vaccination and stamping out programmes are not feasible or sustainable in terms of cost. *Brucella* in milk is killed by pasteurization [13]. In the past, milk borne diseases have been common even in Europe [14]; however pasteurization significantly reduced the incidence. Especially in the city areas of developing countries with lower ratios of animal: human population density compared to corresponding rural areas [2], basic control measures – e.g. boiling, would be effective for reduction of human cases. The practice of boiling milk is common among consumers in Kampala (93%) and milk is usually consumed as ‘milk tea’ [15]. In Kenya, the boiling of milk for preparation of ‘milk tea’ and porridge is also reported to be common [16], [17], but preparation of traditional fermented milk (without boiling the milk) was found to be common among both dairy (31%) and non-dairy farming (22%) households in Kenya; a practice that may present a risk to consumers [17].

The present study in Kampala focuses on unpackaged milk that is informally marketed. Using data obtained from two field surveys (cattle farms and milk shops) together with a medical records survey, we have assessed the risks for consumption of live *Brucella* ingested with raw milk by the urban population. We applied a quantitative risk analysis with stochastic modelling of the dairy value chains flowing into the urban areas of Kampala, using the boiling practice of milk as the inactivation parameter.

The modeling framework was structured as a farm-to-fork microbial food safety risk assessment based on the Codex Alimentarius Commission system [18]. A significant feature of the present model was that stratified dairy value chains were constructed separately and assembled into a dairy value chain risk model, in order to compare the efficacy of the control options. In the value chain model, any increase or decrease of bacterial load was not factored. *Brucella* is an intracellular bacterium that multiplies within macrophages. Macrophages are stable to changes of temperature [19] and acidity [20] and have a long tissue life span under normal circumstances [21]. Even if the infected macrophages are disrupted, the bacteria will survive in milk and cheese, cream, fermented milk and ice cream unless the milk is pasteurized [10], [22]. *Brucellae* are unlikely to increase significantly after being shed in milk and cooled and considering the relatively short time taking from the production to consumption, the natural decrease of *Brucella* in milk was also not factored.

A dose-response model is often used in farm-to-fork risk assessments; however, accurate data for such is often difficult to obtain. In circumstances where a dose-response model is not available, the risk of human cases of food borne illness can be modelled using the level of exposure with the hazard to the population. The U.S. Food and Drug Administration (FDA) Center for Veterinary Medicine (CVM) estimated the risk of human cases of campylobacteriosis caused by fluoroquinolone-resistant *Campylobacter* using a linear population risk model [23]. We can simulate the human health benefit of control in terms of the number of human brucellosis cases avoided under the assumption that the quantity of milk infected with *Brucella* and sold in urban Kampala has a linear relationship with incidence of human brucellosis in the same areas.

## Materials and Methods

### Ethical statement

This study involves an investigation using human medical records as well as blood sampling from cattle. The study protocol was assessed and approved by the Uganda National Council for Science and Technology (UNCST). Access to the anonymized medical records of Mulago Hospital was granted by the Director General of Health Services, Ministry of Health, Uganda. Written consent was not collected from patients because personal data were not recorded in the brucellosis test results and we accessed these records simply to count monthly numbers of the cases diagnosed. Access to farmers and milk sellers for sampling and interviews was granted by the Director Animal Resources, Ministry of Agriculture, Animal Industry and Fisheries. Prior to the milk sampling and interviews with retail milk sellers, wholesalers and boiling centres, and blood sampling and interviews with cattle farmers, the purpose and procedure was explained and informed consent was provided orally; only data on milk production practices and milk sales were collected, no personal data were recorded. The cattle testing was undertaken as a part of the normal veterinary extension services provided to the study communities, and the results were returned to each individual through the veterinary officers, following the normal process of testing and reporting in Uganda.

### Study sites

**Figure 1:**
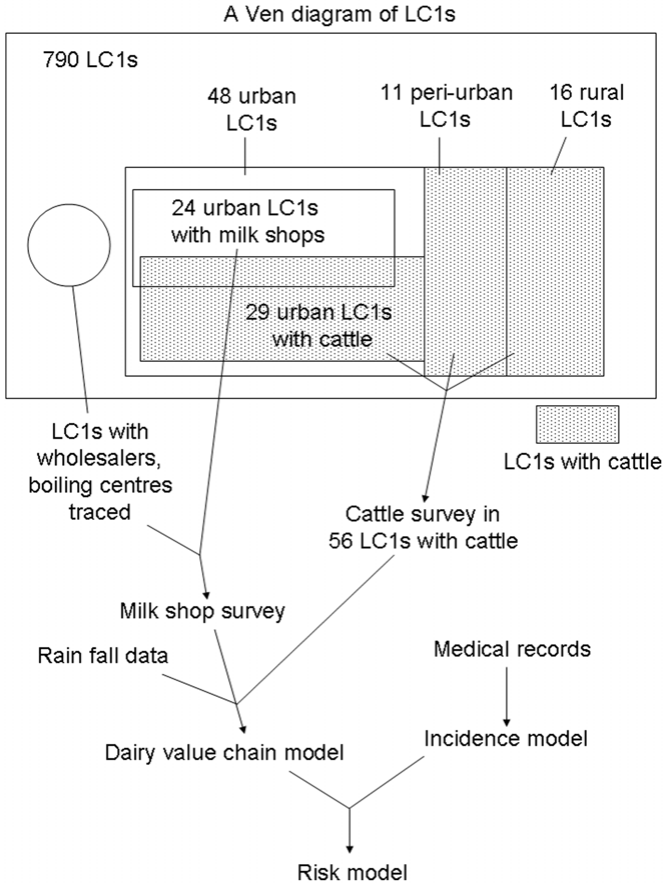
Structure of data sets and a risk model for brucellosis in Kampala. LC1 is Local Council I, the smallest administrative unit of Uganda. The largest rectangular area of the Ven diagram represents all the 790 LC1s in the selected 10 LC3s.

This study was conducted at the Local Council 1 (LC1) level which is the smallest administrative units in Uganda. Uganda has an administrative system consisting of 5 levels: District (LC5), County (LC4), Sub-County (LC3), Parish (LC2) and zone/village (LC1) [24]. The Ven diagram (Figure 1) illustrates the sampling framework. Initially, the sum total of 790 LC1s within the 10 LC3s where more than half of the area was located between 5 to 20 km distances from the Kampala city centroid were listed as an external population. We call the selected areas and the central zones which were surrounded by the study areas the ‘Kampala economic zone,’ since the urban eco-system of Kampala had expanded beyond the demarcation of Kampala District itself. A stratified random sampling was used, where the strata were LC3s and sampling units were LC1s, and 86 LC1s were selected and classified into urban (59 LC1s, not shown in Figure 1), peri-urban (11 LC1s) and rural (16 LC1s) using a published decision tree model based on interviews with LC1 leaders and residents [25]. In urban areas, 48 out of 59 LC1s were selected after excluding LC1s on university/school-owned land (7 LC1s) and very high income residential areas (4 LC1s) in which interviews could not be conducted.

### Cattle farm survey

A survey of cattle keeping farms was conducted in the 56 LC1s (shaded in Figure 1) using multistage random sampling (primary sampling unit: cattle herd, secondary sampling unit: milking cow) during October and November 2007 [26]. WinEpiscope 2.0 was used to calculate the sample size for prevalence estimates [27]. Expected herd prevalence was set at 55.6% based on herd prevalence of brucellosis reported for Mbarara [8], with accepted error and level of confidence set at 5% and 95% respectively. Only milking cows, including cows in dry period were selected as secondary sampling units; bulls, calves and heifers were excluded. The sample size of the secondary sampling unit (milking cow) in each farm was determined at the farm based on a card indicating sample sizes at different herd sizes for disease detection prepared prior to the survey using FreeCalc version 2 (Australian Veterinary Animal Health Services). Sensitivity and specificity were entered as 95.4% and 99.9% respectively [28], [29] as an imperfect test, Buffered antigen plate agglutination test (BPAT), was initially to be used for this study complemented by competitive enzyme-linked immunosorbent assay (CELISA); however we eventually used the CELISA for all samples. The sensitivity, 95.4% was taken from the sensitivity of BPAT (sensitivity 0.954, specificity 0.977) and the specificity, 99.9% was taken from the specificity of CELISA (sensitivity 1.000, specificity 0.999). The estimated animal level prevalence was set at 5%, which is lower or equal to the individual animal prevalences in other studies in the central Uganda [4], [6].

Sera from 425 milking cows in 177 herds were sampled during October and November 2007 and tested for brucellosis using commercial kits of CELISA purchased from the Veterinary Laboratories Agency (Surrey, UK). During the survey, farmers were interviewed for information relating to farming practices, the milking of cows, sales destination and milk yields using a structured questionnaire.

### Milk shop survey

A milk shop survey was conducted using multi-stage cluster sampling (cluster: LC1s, sampling unit: milk sellers) during September and October 2007 during the rainy season. Informal milk sellers were categorized into 5 types prior to the survey: i) milk shops with a bulk cooler, ii) milk shops with a small refrigerator, iii) milk shops without a refrigerator, iv) milk traders/vendors with milk cans on a bicycle and v) roadside milk vendors (as defined in Table 1).

**Table 1:** Definitions of the types of informal-marketed milk sellers seen in Kampala.

**1**	**Milk shop with a bulk cooler**	Milk shops storing milk in a bulk cooler which shape is either box or cylinder. There are two types: wholesaler and retailer.
**2**	**Milk shop with a small refrigerator**	Milk shops storing milk in a small refrigerator.
**3**	**Milk shop without a refrigerator**	Milk shops storing milk in a basin at an ambient temperature.
**4**	**Milk trader with a milk can on a bicycle**	There are three types:1) Those who buy milk at peri-urban farms and sell to contracted individual households and passing trade2) Those who buy at milk boiling centres and sell to contracted individual households and passing trade3) Those who buy milk at wholesaler bulk cooler milk shops and sell to contracted individual households and passing trade or smaller milk shops.
**5**	**Roadside milk vendor**	Milk vendors selling milk at roadside in the early mornings and the evenings. There are three types:1) Those who buy milk at peri-urban farms and sell milk on the roadside in trading centres2) Those who buy milk at boiling centres and sell milk on the roadside in trading centres3) Those who cook milk tea on the roadside (however this type is excluded from the present study since this milk is boiled and therefore not a risk).

In the 24 urban LC1s with milk shops (see Figure 1), all 74 milk shops and 5 roadside milk vendors selling unpackaged milk (liquid milk which is not packaged, including both pasteurised and non-pasteurised milk) were interviewed using a structured questionnaire to identify the informal dairy value chains (a value chain is a chain of activities, or elements of a supply chain that together make up a complete system – in our case the supply of milk to consumers from the site of production). From these, milk was sampled from 59 shops and all 5 roadside vendors. Questions included the source of milk, average quantity of milk sales per day during working days, number of working days per week, boiling practices, mode of milk transportation and destination of milk (retail sales and/or vendors). The wholesalers and boiling centres in urban Kampala (Figure 1) which were traced back as the source of milk for these interviewed milk sellers and vendors were visited and interviewed using the same questionnaire and milk was sampled in the same manner. Milk was sampled using a disposable Pasteur pipette and transferred into an Eppendorf tube. The milk sample was immediately stored in a cool box and carried to the Central Laboratory in the Department of Veterinary Medicine, Faculty of Veterinary Medicine, Makerere University, Uganda where samples were stored at −20°C. In total, 135 sellers were interviewed and 117 unpackaged milk samples were collected (Table 2). Twenty-one sellers did not provide samples after interview because they had sold out of their product.

**Table 2:** The numbers of milk sellers interviewed, purchasing raw milk, boiling milk and sampled.

Type of milk sellers	Interviewed	Purchasing raw milk	Boiling	Sampled
Boiling centre	5	5	5	4
Wholesaler shop with bulk coolers	30	30	0	27
Retail shop with a bulk cooler	17	17	0	17
Shop with a small refrigerator	52	48	2	39
Shop without a refrigerator	4	3	1	3
Vendor with a milk can on a bicycle	22	21	0	22
Roadside vendor	5	3	0	5
Total	135	127	8	117

Sampled milk was tested at the Department of Molecular Microbiology, Faculty of Veterinary Medicine, Makerere University using an indirect enzyme-linked immunosorbent assay (IELISA) [28] kit purchased from the Veterinary Laboratory Agency, UK. We did not use the CELISA for milk testing because the commercial kits for milk were not available; we account for the known ‘imperfect’ nature of the IELISA test in our analysis [30]. Optical density (OD) values were read using a calibrated ELISA plate reader, and samples were classified as either brucellosis positive or negative.

### Estimation of annual human brucellosis incidence rate in urban Kampala

The serological test results for human brucellosis screening, routinely conducted in the Department of Microbiology, Mulago National Referral Hospital, Kampala, between June 2004 and May 2006 were digitised. Mulago Hospital uses the plate agglutination test (sensitivity and specificity are 0.771 and 0.960) [28]. Patients are tested in the Outpatient Department when febrile episodes are suspected as attributable to brucellosis. The number of monthly diagnoses showed unusual seasonality but the technical staff in the Department of Microbiology explained that this is due to a limitation in the number of test kits with which the hospital is supplied. Consequently, only when the number of tests per month exceeded 20 (i.e. indicating that test kits were freely available) did we use the data to estimate the distribution of the annual true incidence among outpatients of Mulago Hospital. Thirteen out of the 24 monthly periods met this criterion (which avoided test availability bias) and analysis was undertaken using a bootstrapping technique in @Risk version 5.0 (Palisade Decision Tools).

To estimate the annual incidence of human brucellosis in urban areas of Kampala, the annual diagnoses in all the large hospitals in urban Kampala was estimated extrapolating the Mulago Hospital data to other large hospitals in Kampala based on the numbers of hospital beds. As the estimated annual diagnoses include patients from peri-urban and rural areas, the annual urban brucellosis incidence was estimated using a reported proportion of urban patients among total diagnoses in Mulago Hospital [2]. The incidence model (Figure 1) was run for 10,000 iterations using Monte Carlo simulation in @Risk. The population living in the urban parishes classified based on a previous study [25] was obtained from the Ugandan Bureau of Statistics. The estimation of annual incidence rate was calculated by dividing the annual urban incidence by this urban population.

### Development of the dairy value chain model

**Figure 2:**
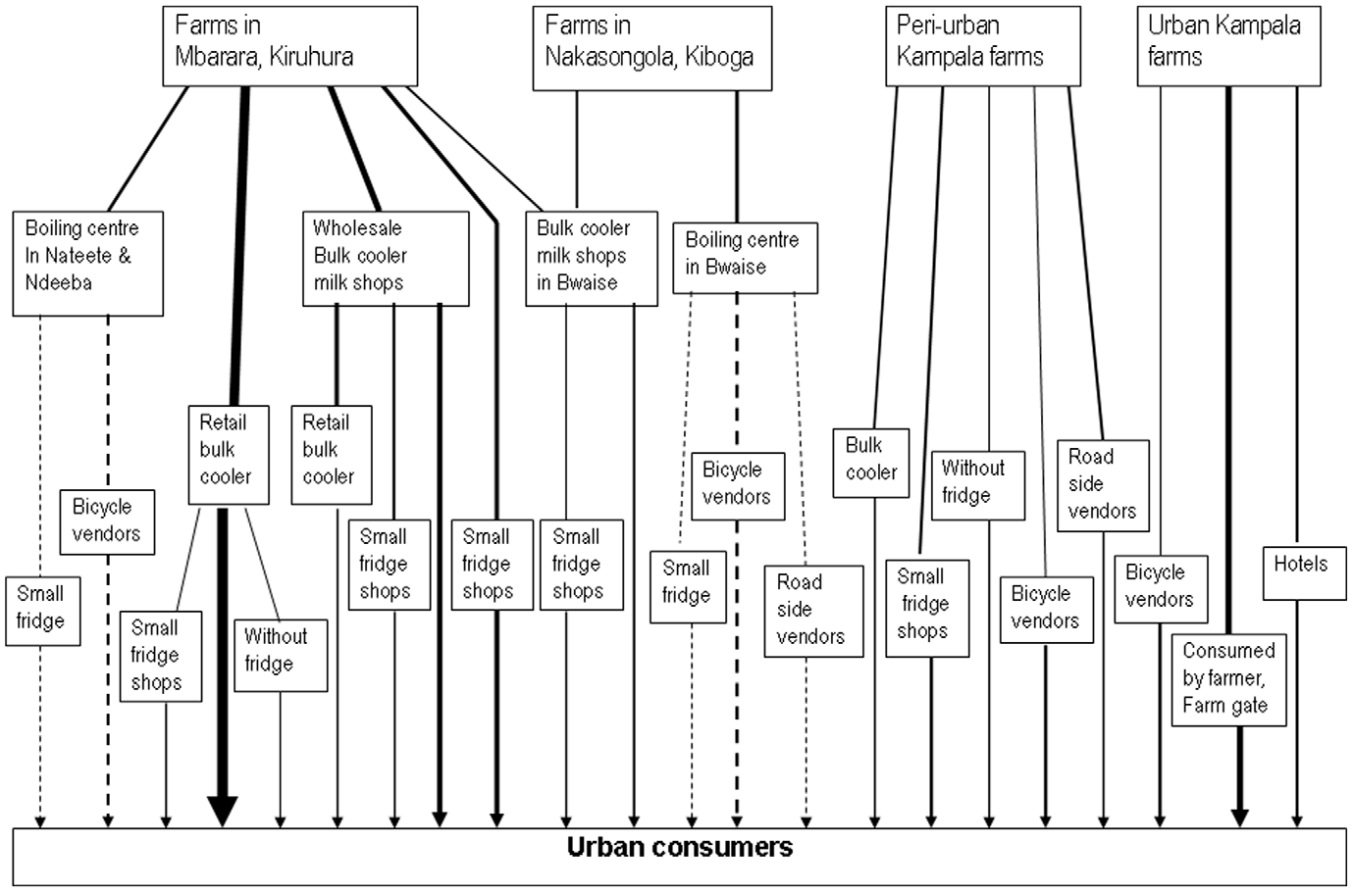
Diagram showing dairy value chain in urban areas of Kampala. Solid lines show raw milk distribution although some proportion is boiled. Dashed lines show distribution of treated milk from boiling centres. The width of lines represents a variation in quantity of milk distributed.

**Figure 3:**
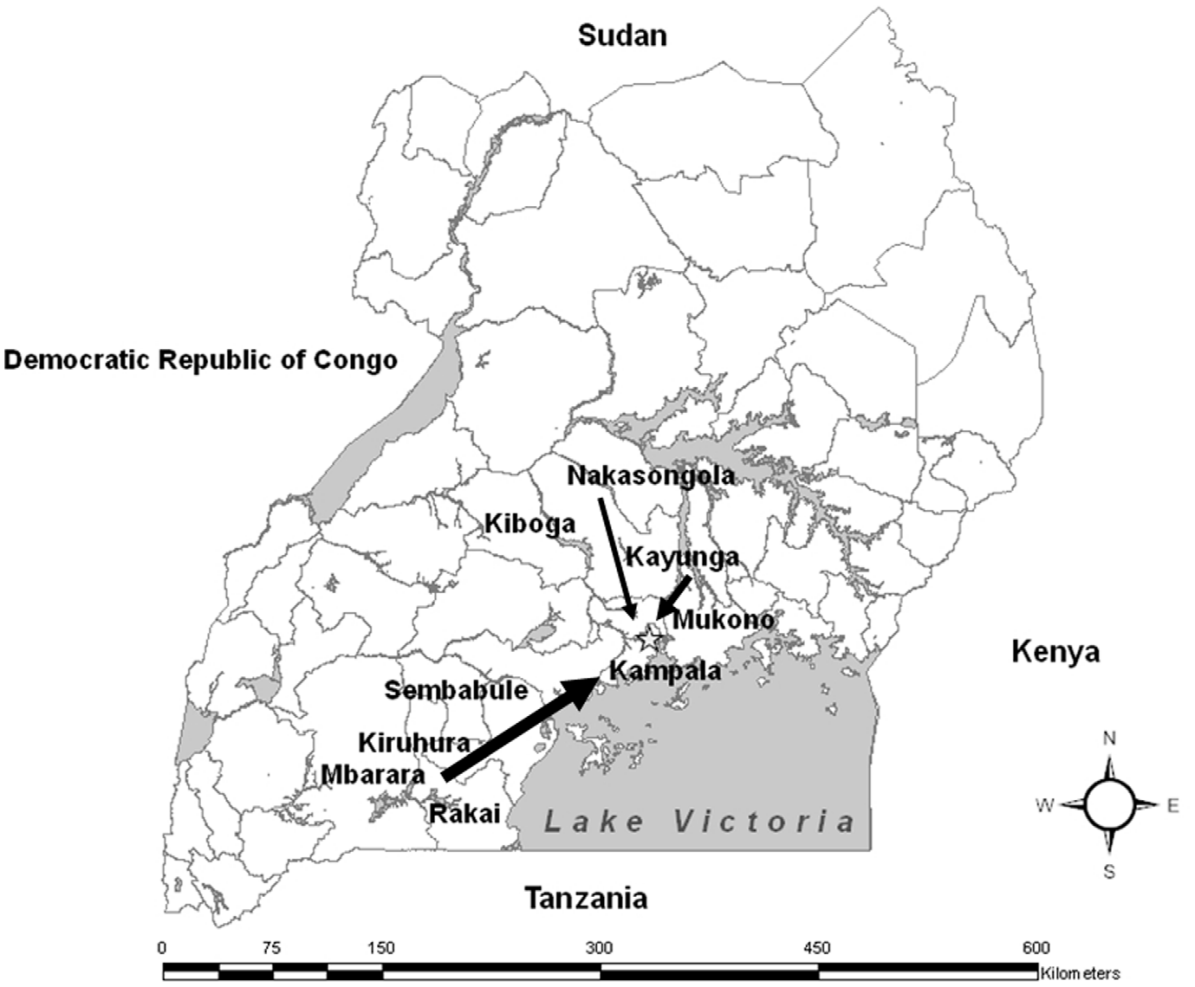
Map showing distribution of milk from production areas to Kampala. The width of arrows represents the quantities of milk transported.

A dairy value chain model for brucellosis was developed synthesizing the data from the cattle farm survey and the milk shop survey, based on the identified dairy value chain (Figure 1, Figure 2). The source of milk was categorized as one of: Mbarara dairy production areas, Nakasongola/Luweero production areas, peri-urban and urban areas of Kampala (Figure 2, Figure 3).

The value chain model consisted of milk quantity, infection rate and inactivation parameters. In a continuing value chain from production areas to consumers, the infection rate at the origin was multiplied by 1- the probability of boiling at the origin node of the pathway to calculate the infection rate at the end of pathway and then it was fed into the next pathway as the infection rate at origin and continued until the pathway reached the consumers. The daily quantities of *Brucella* infected milk reaching consumers through all the risk pathways were summed to calculate the total daily quantity of infected milk, which was assumed to have a positive linear relationship with the urban incidence of human brucellosis (see File S1 for details).

The quantities of milk sales obtained from surveys were these for the rainy season. Milk production correlates with the availability of grass which correlates with rainfall. Therefore, annual average quantities of milk sales were estimated by multiplying the quantities with the ratio of annual average rainfall and rainfall in rainy season taking the following step. The annual average quantities were estimated for two regions: Mbarara production areas, and the other areas using rainfall data in Mbarara and Namulonge (located in northern Kampala), respectively. For these estimations, quantities obtained from surveys in rainy season were divided by mean bootstrap of September and October monthly rainfall records between 1999 and 2005 and then multiplied by mean bootstrap of all monthly rainfall records of the same years [31] (see File S1 for details).

The infection rates in Mbarara production areas and Nakasongola, Luweero production areas were estimated using Bayesian inference. Since the milk IELISA is an imperfect test, we used estimates of sensitivity 0.953 and specificity 0.951 for pooled bulk milk samples from the published literature [30]. IELISA is an antibody test and a test positive does not necessarily mean that *Brucella abortus* is found in the milk; however due to the time limitations, the commercial kits were used. The results of 66 samples sourced from Mbarara production areas (8/57 IELISA positive) and Nakasongola, Luweero production areas (2/9 IELISA positive) were used in the model, regardless of the type of milk sellers. CELISA results of cattle sera were used for the estimation of milk infection rates for milk sourced from urban and peri-urban areas of which the majority sold by vendors with a milk can on a bicycle. In urban areas, the herd prevalence was 7.4% (4/54). In peri-urban areas, herd prevalence of brucellosis in large herds (3/6, 50%) which cattle graze in free range was higher than that of small herds (4/117, 3.4%, publication in preparation). Therefore quantities of infected milk produced by peri-urban large herds and small herds were modeled separately and summed to obtain the quantity of infected milk produced in peri-urban areas and sold to urban areas. Each farm was modeled to have the uncertainty in milk yield and the status of being infected or not given by the CELISA results.

Inactivation (boiling) parameters were modelled stochastically based on the results from interviews, except boiling centres (the probability: 1), sellers purchasing from boiling centres (0, sellers never boil pasteurized milk), vendors with a bicycle and milk shops with a bulk cooler (0), roadside milk vendor (0), milk shops with a bulk cooler (0) and large hotels (1) which were modelled deterministically, as the uncertainty was judged to be negligible.

### Exposure assessment

The dairy value chain model was run for 10,000 iterations of Monte Carlo simulation in @Risk and the probability distributions of the total quantity of informally marketed milk, the total quantity of informally marketed milk infected with brucellosis, and the overall milk infection rate at purchase were obtained. Table 3 shows summarized parameters used in the risk model. Separation of variability from uncertainty was done by changing variability distributions into point estimates because the model was too complex to model uncertainty and variability distinguished. Sensitivity analysis was done for uncertainty and variability parameters used simulating 105 times with 1000 iterations each in @Risk simulation.

**Table 3:** Summarized parameters used in the model and their statistical descriptions.

Summarized parameters	Statistics (90% CI)	Distributions used	Uncertainty/variability
Total quantity of daily milk sales in urban Kampala	148.9 t (109.7–200.0)	Bootstrap simulation	Variability
Milk infection rate in Mbarara	0.115 (0.039–0.206)	Bayesian inference with non informative prior (1,1) and Binomial likelihood distribution adjusted with sensitivity and specificity of IELISA	Uncertainty
Milk infection rate in Nakasongola	0.250 (0.050–0.508)	Bayesian inference with non informative prior (1,1) and Binomial likelihood distribution adjusted with sensitivity and specificity of IELISA	Uncertainty
Milk infection rate in urban farms	0.075 (0.010–0.166)	Simulated total quantity of infected milk/simulated total milk production, using bootstrap and Binomial distribution	Uncertainty + variability (milk quantity)
Milk infection rate in peri-urban farms	0.253 (0.086–0.426)	Sum of total quantities of infected milk produced by small and large scale farms/sum of total milk productions by small and large scale farms, using bootstrap and Binomial distribution	Uncertainty + variability (milk quantity)
Boiling practice	Probabilities in each type of milk sellers were used	Beta distribution and point estimates	Uncertainty
Annual average/rainy season rainfall ratio in Mbarara	0.564 (0.457–0.694)	Bootstrap simulation of 7 years data (1999–2005)	Variability
Annual average/rainy season rainfall ratio in Namulonge	0.730 (0.596–0.880)	Bootstrap simulation of 7 years data (1999–2005)	Variability
Human brucellosis incidence	1009 (929–1082)	Beta distribution, Binomial distribution using adjusted prevalence with sensitivity and specificity of test, bootstrap	Uncertainty

As the model is complex, all the individual parameters were presented in the Annex.

### Hazard and risk characterization

Hazard characterization was not undertaken quantitatively because parameters describing the dose-response relationship for brucellosis were not available. In a risk assessment, annual incidence, as a characteristic of the risk, is usually calculated using the exposure to the hazard and the dose-response relationship. However, the characteristics of the risk were expressed as the annual incidence and the annual incidence rate of human brucellosis using the incidence model mentioned previously, due to this limitation.

### Sources of infected milk with *Brucella abortus*

By changing the inactivation parameters of the risk pathways sourced from each production areas and each type of milk sellers, and simulating the reduction in the proportion of milk infected, the contributions of these areas and sellers in risk distribution to the urban population were estimated. Monte Carlo simulation was run for 10,000 iterations each for the estimation.

### Risk mitigation simulations by control options

Risk mitigation simulations were conducted under the assumption that there is a linear correlation between the annual average daily quantity of infected milk sold in urban Kampala (*Vi*) and human brucellosis incidence in the city (*λi*) in year *i* with the population-based dose-response parameter *K*.
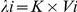



Therefore the future human brucellosis incidence of year *j* after a control option is implemented (*λj*) was simulated for the predicted annual average daily quantity of infected milk sold in urban Kampala (*Vj*) as below.
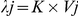



Eight possible control options were prepared based on the dairy value chain model (Table 4). Related inactivation (boiling) parameters were changed to 0.9, when the parameters were lower than that in the original model, assuming 90% of enforcement was achieved for each scenario (i.e. when inactivation parameter was 1, it was kept intact), or 90% of milk sales was allocated to the alternative sellers who were thought to take it over. For the options, banning milk sales by traders with milk cans on a bicycle, roadside vendors and milk shops without refrigerator, the milk sales was modelled to be taken over by milk shops with a bulk cooler, as it was thought to be the most likely option. For the option of banning urban dairy farming, urban milk production was modelled to be taken over by wholesalers transporting milk from Mbarara. For the option of banning milk sales at the farm gate, the milk sales was modelled to be taken over by vendors with milk cans on a bicycle. In other words, we modelled the most likely alternative sources of milk for consumers given our hypothetical interventions. The reduction rate of infected milk (the quantity of milk avoided the contamination after the implementation of control option was divided by the quantity of contaminated milk before the implementation), the annual incidence avoided and the annual incidence rate after control option was implemented were simulated with 10,000 iterations of Monte Carlo simulation in @Risk for each scenario to show the human health benefits. Necessary inputs, challenge, feasibility, sustainability and negative impact of these options were assessed qualitatively to find the best control option of human brucellosis in urban areas of Kampala.

**Table 4:** Assessment of control options by simulations assuming 90% of enforcement was achieved.

Control options	Reduction rate (percentage)	Incidence avoided	Inputs	Feasibility	Negative impact	Assessment
Construct a boiling centre in Mbarara	47.4 (21.6–70.1)	477 (224–710)	A boiling centre, legislation, fuel	Middle-high	Price up	Recommendable
Construct boiling centres in peri-urban Kampala	82.0 (71.0–89.0)	825 (702–926)	Boiling centres, legislation, fuel	Middle	Price up, peri-urban soon becomes urban	Recommendable
Enforce milk shops to boil milk or to purchase boiled milk	56.6 (35.9–75.0)	568 (361–759)	Legislation, fuel, facility, enforce	Very low	Price up, corporation less likely to be given	Not recommendable
Ban of farm gate milk sales	0 (0.0–0.0)	0 (0–0)	Legislation, enforcement	Low	Alternative sellers may not boil	Not recommendable
Ban of urban dairy farming	−11.8 (−19.4–−5.4)	−118 (−196–−54)	Legislation, enforcement	Middle	Livelihood of urban farmers, milk supply shortage	Not recommendable
Ban of milk sales by vendors with a bicycle	0.0 (0.0–0.0)	0 (0–0)	Legislation, enforcement	High	Livelihood of vendors, alternative sellers may not boil	Not recommendable
Ban of roadside milk sales	0 (0.0–0.0)	0 (0–0)	Legislation, enforcement	High	Livelihood of vendors, alternative sellers may not boil	Not recommendable
Ban of milk sales at shops without refrigerators	−0.4 (−0.9–−0.1)	−4 (−10–−1)	Legislation, enforcement	High	Livelihood of vendors, alternative sellers may not boil	Not recommendable

Within () is 90% confidence interval.

## Results

### Risk assessment

On average, 148,924 litres (90% CI: 109,669–200,011) of unpackaged milk was consumed by the urban population each day and 12.6% (90% CI: 6.8–18.9) of the milk was estimated to be contaminated with *Brucella*. Separation of variability did not change the uncertainty of milk contamination rate with *Brucella*: 12.6% (90% CI: 7.0–18.9). The most sensitive uncertainty parameters were infection rates in Mbarara, peri-urban areas and urban areas in this order (Table 5). The risk of infection with brucellosis in urban areas of Kampala was expressed as the estimation of annual incidence of human brucellosis, 1009 (90% CI: 929–1,082) and the annual incidence rate, 5.8 (90% CI: 5.3–6.2) per 10,000 people.

**Table 5:** Sensitivity analysis results in the order of the sensitivity to the probability of purchasing infected milk with *Brucella*.

Order	Parameters	Values with 50^th^, 1^st^ & 99th percentiles	Mean probability of purchasing infected milk at the values
1	Milk infection rate in Mbarara	0.094 (0.063–0.125)	0.114 (0.096–0.133)
2	Milk infection rate in PU areas	0.162 (0.109–0.215)	0.112 (0.102–0.121)
3	Milk infection rate in urban areas	0.165 (0.111–0.219)	0.140 (0.133–0.148)
4	Sales of milk produced in PU and sold to urban areas by vendors with a bicycle (t/day)	38.9 (26.2–51.6)	0.135 (0.127–0.141)
5	Sales of milk produced and sold in urban areas by vendors with a bicycle (t/day)	38.3 (25.8–50.8)	0.125 (0.122–0.128)
6	Sales of milk produced in Mbarara and sold to retail milk shops with a bulk cooler (t/day)	130.2 (87.7–172.7)	0.127 (0.125–0.129)

### Source of the risk

**Figure 4:**
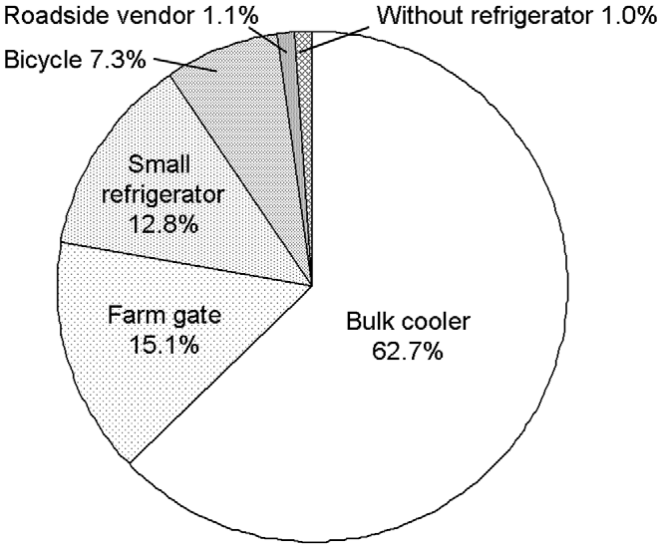
Sources of infected milk with *Brucella abortus* by the types of milk sellers and the proportions of milk sold.

When different types of milk sellers were compared as to which types are contributing to human brucellosis incidence, milk shops with a bulk cooler sold the largest proportion of infected milk (50.5%, 90% CI: 26.4–73.0, Figure 4). Vendors with a milk can on a bicycle were the second largest source (27.7%, 90% CI: 10.7–47.1) and shops with a small refrigerator the third (11.9%, 90% CI: 8.1–16.5).

**Figure 5:**
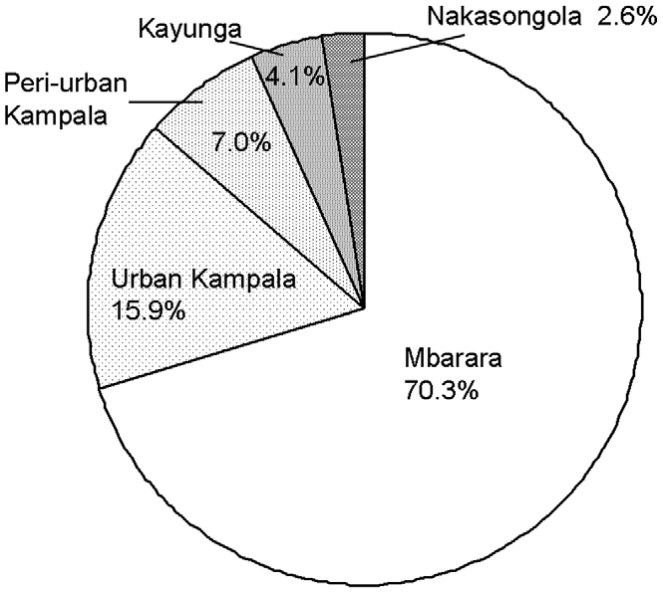
Sources of infected milk with *Brucella abortus* by the production areas and the proportions of milk distributed.

Among the 4 production areas that were categorized (Mbarara, Nakasongola/Luweero, peri-urban and urban production areas), the largest proportion (52.5%, 90% CI: 24.4–77.1) of infected milk was distributed out of the Mbarara production area and the second largest source was the peri-urban areas (35.5%, 90% CI: 13.0–60.2, Figure 5). Urban farms distributed 9.0% (90% CI: 1.2–21.0) of infected milk and Nakasongola/Luweero production areas distributed the lowest proportion of infected milk (3.0%, 90%CI: 0.4–7.7).

### Risk mitigation simulations

The reduction of risk would be greatest (82.0%, 90% CI: 71.0–89.0) by constructing boiling centres in peri-urban areas and enforcing rules requiring traders from production areas and peri-urban dairy farms to sell milk exclusively to them for treatment and resale. However, the cost of the construction, legislation and enforcement present major challenges and the retail price of milk would likely increase, making such measures unpopular with the consumers (Table 4). With this optimal option, the annual incidence rate reduces to 1.0 (90% CI: 0.6–1.7) per 10,000 people and 825 (90% CI: 702–926) human brucellosis cases would be avoided annually.

The next greatest reduction (56.6%, 90% CI: 35.9–75.0) would be achieved by enforcing rules that require milk shops to boil milk or to sell only boiled milk. However this option would place an unrealistic financial burden on suppliers who lack the space for the installations required.

Constructing a boiling centre in the Mbarara production areas and requiring traders and farmers to sell milk to this centre is an option that would reduce risk by 47.4% (90% CI: 21.6–70.1), resulting in an estimated annual incidence rate 2.7 (90% CI: 1.3–4.0) per 10,000 people and 477 (90% CI: 224–710) cases would be avoided annually. This option would be efficient - since most milk is sold in the shops that posses a bulk cooler, boiling would eliminate the most significant source of risk.

Prohibitive measures would not reduce risk but would have negative impacts on the livelihoods of many farmers and milk sellers. Even worse, banning urban farming and milk sales at shops without refrigerators would increase the risks by 11.8% (90% CI: 5.4–19.4) and 0.4% (90% CI: 0.1–0.9), respectively.

## Discussion

The present study presents the first stochastic risk assessment for milk-borne human brucellosis infection in Kampala. Through this assessment, the annual human brucellosis incidence rate was estimated and the best control options for the disease, in this case, constructing boiling centres in Mbarara production area and/or peri-urban areas, is recommended.

Pasteurization or boiling of milk is the most basic control of brucellosis in humans. It cannot be the absolute solution to prevent this disease in rural parts of an endemic area where people live close to diseased livestock and wildlife. However in urban areas of Kampala, animal density against human density is known to be significantly lower than in peri-urban and rural areas [2]. This suggests that the basic control, boiling milk, would be effective to reduce incidence among a population who do not contact animals but consume dairy products purchased in informal markets. Milk pasteurization is also an integrated control measure for multiple pathogens. Raw milk may transmit not only brucellosis but also many other diseases to humans, such as bovine tuberculosis [8], *E. coli* O157 [17], listeriosis, salmonellosis [32], campylobacteriosis [33] and scarlet fever [34]. The presence of zoonotic pathogens in raw milk [35] does not necessarily imply that people become ill by consuming it [36], especially in the countries where milk pasteurization is implemented. However in Uganda where a cattle herd prevalence of *Mycobacterium bovis* was reported to be 74.1% in Mbarara [8], milk pasteurization would surely have a multiple impact against zoonoses [37]. An information campaign is another basic and effective measure which should be considered in brucellosis control in urban areas.

Risk assessment frameworks have greatly contributed to improved food safety in developed countries and these frameworks should provide a useful decision support tool to policy makers in developing countries as well. However the high quality of data required for inputs into farm-to-fork microbial food safety risk assessments are often lacking. Nevertheless, here we have shown that by using a combination of field surveys, hospital record data and modelling, that such assessments are possible in developing country settings. The design of the model which dealt each value chain separately, rather than estimating only the overall risk, made it possible to simulate the reduction of risks by each control option. Sensitivity analysis showed that the milk infection rate in Mbarara is the most sensitive parameter. The present study used the milk IELISA, an antibody test. Sensitive tests detecting a pathogen such as polymerase chain reaction in milk may be better to be used for the future updates. Boiling milk obviously changes the risk greatly but we did not model it stochastically for shops with bulk coolers as all the shops did not boil, according to the interviews.

The true urban brucellosis incidence rate was estimated adjusting the reported incidence with the sensitivity and specificity of the plate agglutination test and the reported proportion of urban dwellers among all brucellosis cases in Mulago Hospital. However, the estimates of incidence rate, described here, may still greatly underestimate the true figure since this is based on simulated diagnoses in large hospitals and ignores the patients who go to small clinics (where they are unlikely to be appropriately tested) or who don't present for testing at all.

The construction of boiling centres in the Mbarara production area and/or peri-urban areas were the most favourable option since this would greatly reduce risk while not affecting livelihoods for people associated with the milk production and dairy value chain in the city. It is important to permit the urban poor the widest possible range of opportunities [38]. Proposals to ban milk vendors/traders (milk can on a bicycle) within urban areas in Kampala would not significantly reduce the risks but would deleteriously affect the livelihoods of a large number of traders. Banning urban dairy farming would likely actually increase the risks because of the high prevalence of brucellosis among cattle herds in the dairy production areas. The DDA, Uganda has had a significant impact on dairy hygiene by the introduction of cooling trucks to transport milk from dairy production areas to Kampala City (since 2006), and was planning further developments of the milk cold chain by the provision of milk cooling centres in peri-urban Kampala. However, the DDA were not promoting the practice of boiling milk in the informal market.

While the construction of boiling centres would seem like a reasonable public health measure, the construction of boiling centres in peri-urban areas would be costly. The second issue is the rapid expansion of the urban areas [25]. Policy makers need to plan strategically as peri-urban areas are rapidly becoming urban. Facilities should be designed to relocate easily or additional plants may be best located outside the city. Construction of boiling centres in Mbarara for example, would not be affected by the urbanisation of Kampala because Mbarara is far enough from Kampala; however their impact would be less than constructing them in peri-urban areas. The third issue is the compliance with such control programmes from both traders and consumers.

In conclusion, the risk of human brucellosis in urban areas through consumption of informally marketed raw milk could be greatly reduced by construction of boiling centres. This measure would have multiple impacts on many hazardous zoonoses transmitted through milk, and needs to be considered urgently. It is important to raise awareness of this public health problem among policy makers and to communicate risks to consumers in urban areas of Kampala.

Further qualitative studies on the perception and behaviour of milk sellers, traders and consumers related with dairy hygiene and incentives to comply with disease control programmes are needed. Urban dairy farmers and consumers in Kampala are known to be taking risk mitigation strategies by themselves [15]. Entry points for sustainable and feasible disease control messages need to be identified, and detailed economic studies on the options that would be adopted by producers, traders and consumers would be useful.

## Supporting Information

File S1 Detailed structure of the risk model.
(0.16 MB DOC)
