# Supplementary material for: How Human Brucellosis Incidence in Urban Kampala Can Be Reduced Most Efficiently? A Stochastic Risk Assessment of Informally-Marketed Milk
Source: PLoS One. 2010 Dec 1;5(12):e14188. doi: 10.1371/journal.pone.0014188 (PMC2995731; doi:10.1371/journal.pone.0014188)
Supplement: File S1 — Detailed structure of the risk model. (0.16 MB DOC) [file pone.0014188.s001.doc]

**Annex 1a Structure of brucellosis dairy value chain** model

| Pathway | L/day  (rainy) | L/day  (average) | Infection rate at origin | Boiling at origin | Infection rate at the end | Litre contaminated |
| --- | --- | --- | --- | --- | --- | --- |
| **A. From Mbarara production areas to;**  A1. Boiling centres in Nateete and Ndeeba  A2. Retail bulk cooler milk shops  A3. Wholesale shops in Nateete, Kitintale  A4. Small refrigerator milk shops  A5. Bulk cooler shops in Bwaise, Kawempe | qrA1  qrA2  qrA3  qrA4  qrA5 | qaA1  qaA2  qaA3  qaA4  qaA5 | irMb  irMb  irMb  irMb  irMb | bMb  bMb  bMb  bMb  bMb | irA1  irA2  irA3  irA4  irA5 | qcA1  qcA2  qcA3  qcA4  qcA5 |
| From A1 to;  A1.1. Small refrigerator milk shops  A1.2. Vendors with a can on a bicycle | qrA1.1  NA | qaA1.1  qaA1.2 | irA1  irA1 | bA1  bA1 | irA1.1  irA1.2 | qcA1.1  qcA1.2 |
| From A1.1 to **consumers**  From A1.2 to **consumers** | NA  NA | qaA1.1  qaA1.2 | irA1.1  irA1.2 | bA1.1  bA1.2 | irA1.1c  irA1.2c | qcA1.1c  qcA1.2c |
| From A2 to;  A2.1. Small refrigerator milk shops  A2.2. **Consumers**  A2.3. Milk shops without a refrigerator | qrA2.1  NA  qrA2.3 | qaA2.1  qaA2.2  qaA2.3 | irA2  irA2  irA2 | bA2  bA2  bA2 | irA2.1  irA2.2  irA2.3 | qcA2.1  qcA2.2c  qcA2.3 |
| From A2.1 to **consumers**  From A2.3 to **consumers** | NA  NA | qaA2.1  qaA2.3 | irA2.1  irA2.3 | bA2.1  bA2.2 | irA2.1c  irA2.3c | qcA2.1c  qcA2.3c |
| From A3 to;  A3.1. Retail bulk cooler milk shops  A3.2. Small refrigerator milk shops  A3.3. **Consumers** | qrA3.1  qrA3.2  NA | qaA3.1  qaA3.2  qaA3.3 | irA3  irA3  irA3 | bA3  bA3  bA3 | irA3.1  irA3.2  irA3.3 | qcA3.1  qcA3.2  qcA3.3c |
| From A3.1 to **consumers**  From A3.2 to **consumers** | NA  NA | qaA3.1  qaA3.2 | irA3.1  irA3.2 | bA3.1  bA3.2 | irA3.1c  irA3.2c | qcA3.1c  qcA3.2c |
| From A4 to **consumers** | NA | qaA4 | irA4 | bA4 | irA4c | qcA4c |

**Annex 1b Structure of brucellosis dairy value chain** model

| Pathway | L/day  (rainy) | L/day  (average) | Infection rate at the origin | Boiling | Infection rate at the end | Litre contaminated |
| --- | --- | --- | --- | --- | --- | --- |
| **B. From Nakasongola, Luweero to;**  B1. Bulk cooler shops in Bwaise, Kawempe  B2. Boiling centre in Bwaise | qrB1  qrB2 | qaB1  qaB2 | irNL  irNL | bNL  bNL | irB1  irB2 | qcB1  qcB2 |
| From B1 to;  B1.1. Small refrigerator milk shops  (joined with A5)  B1.2. **Consumers** | qrB1.1  NA | qaB1.1  qaB1.2 | irB1  irB1 | bB1  bB1 | irB1.1  irB1.2 | qcB1.1  qcB1.2c |
| From B1.1 to **consumers** | NA | qaB1.1 | irB1.1 | bB1.1 | irB1.1c | qcB1.1c |
| From B2 to;  B2.1. Vendors with a can on a bicycle  B2.2. Small refrigerator milk shops  B2.3. Roadside milk vendors | NA  qrB2.2  qrB2.3 | qaB2.1  qaB2.2  qaB2.3 | irB2  irB2  irB2 | bB2  bB2  bB2 | irB2.1  irB2.2  irB2.3 | qcB2.1  qcB2.2  qcB2.3 |
| From B2.1 to **consumers**  From B2.2 to **consumers**  From B2.3 to **consumers** | NA  NA  NA | qaB2.1  qaB2.2  qaB2.3 | irB2.1  irB2.2  irB2.3 | bB2.1  bB2.2  bB2.3 | irB2.1c  irB2.2c  irB2.3c | qcB2.1c  qcB2.2c  qcB2.3c |
| **C. From peri-urban Kampala farms to;**  C1. Retail bulk cooler milk shops  C2. Small refrigerator milk shops  C3. Milk shops without a refrigerator  C4. Roadside vendors  C5. Vendors with a can on a bicycle | qrC1  qrC2  qrC3  qrC4  qrC5 | qaC1  qaC2  qaC3  qaC4  qaC5 | irPU  irPU  irPU  irPU  irPU | bPU  bPU  bPU  bPU  bPU | irC1  irC2  irC3  irC4  irC5 | qcC1  qcC2  qcC3  qcC4  qcC5 |
| From C1 to **consumers**  From C2 to **consumers**  From C3 to **consumers**  From C4 to **consumers**  From C5 to **consumers** | NA  NA  NA  NA  NA | qaC1  qaC2  qaC3  qaC4  qaC5 | irC1  irC2  irC3  irC4  irC5 | bC1  bC2  bC3  bC4  bC5 | irC1c  irC2c  irC3c  irC4c  irC5c | qcC1c  qcC2c  qcC3c  qcC4c  qcC5c |

**Annex 1c Structure of brucellosis dairy value chain** model

| Pathway | L/day  (rainy) | L/day  (average) | Infection rate at the origin | Boiling | Infection rate at the end | Litre contaminated |
| --- | --- | --- | --- | --- | --- | --- |
| **D. From urban Kampala farms to;**  D1. Vendors with a can on a bicycle  D2. Home consumption, farm gate  D3. Hotels | qrD1  qrD2  qrD3 | qaD1  qaD2  qaD3 | irUr  irUr  irUr | bUr  bUr  bUr | irD1  irD2  irD3 | qcD1  qcD2  qcD3 |
| From D1 to **consumers**  From D2 to **consumers**  From D3 to **consumers** | NA  NA  NA | qaD1  qaD2  qaD3 | irD1  irD2  irD3 | bD1  bD2  bD3 | irD1c  irD2c  irD3c | qcD1c  qcD2c  qcD3c |

**Annex 2 Infection rate at the end of each risk pathway and quantity of contaminated milk passing through each risk pathway**

| Notation | Description | Estimation |
| --- | --- | --- |
| ir….  ir….c  qc….  qc…c | Infection rate at the end of the pathway  Infection rate of milk consumer purchases  Quantity of milk contaminated  Quantity of milk contaminated sold to consumers | Infection rate at origin * (1-probability of boiling)  Above estimation reached to the consumer node  Annual average quantity per day (qa…) * infection rate at the end of pathway (ir…)  Above estimation reached to the consumer node |

**Annex 3** Daily milk quantities passing through risk pathways in rainy season

| Notation | Estimation (Litre) | Source |
| --- | --- | --- |
| qrA1  qrA2  qrA3  qrA4  qrA5  qrA1.1  qrA2.1  qrA2.3  qrA3.1  qrA3.2  qrB1  qrB2  qrB1.1  qrB2.2  qrB2.3  qrC1  qrC2  qrC3  qrC4  qrC5  qrD1  qrD2  qrD3 | (4500*2+5000*5)/7  ∑ bootstrap*790/87, *n*=17  ∑ bootstrap, *n*=15  ∑ bootstrap*790/87, *n*=9  ∑ bootstrap, *n*=10  Single bootstrap of all shops with a small refrigerator*790/87  ∑ bootstrap*790/87  Single bootstrap of all shops without a refrigerator*790/87  300*790/87  ∑ bootstrap*790/87, *n*=9  ∑ bootstrap, *n*=5  7680  ∑ bootstrap*790/87, *n*=11  Single bootstrap of all shops with a small refrigerator*790/87  ∑ bootstrap of all roadside vendors*790/87, *n*=2  150*790/87  ∑ bootstrap *790/87, *n*=14  ∑ bootstrap of all shops without a refrigerator*790/87, *n*=2  ∑ bootstrap of all roadside vendors*790/87, *n*=2  PUprdUr – qrC1 - qrC2 - qrC3 - qrC4  ∑ bootstrap of herd level milk yielding*UrFrc, *n*=2  Urprd – qrD1 - qrD3  78*UrFrc | Milk shop interviews, 4 boiling centres, Nateete boiling centre operates 500L/day, 5days a week  Milk shop interviews, 17 shops  Milk shop interviews, 15 shops  Milk shop interviews, 9 shops  Milk shop interviews, 10 shops  Milk shop interviews, 52 shops  Milk shop interviews, 4 shops  Milk shop interviews, 4 shops  Milk shop interviews, 1 shop  Milk shop interviews, 9 shops  Milk shop interviews, 5 shops  Milk shop interview, 1 boiling centre  Milk shop interviews, 11 shops  Milk shop interviews, 52 shops  Milk shop interviews, 5 roadside vendors  Milk shop interview, 1 shop  Milk shop interviews, 14 shops  Milk shop interviews, 4 shops  Milk shop interviews, 5 roadside vendors  Cattle survey, 42 peri-urban farms  Cattle survey, 54 urban farms  Cattle survey, 54 urban farms  Cattle survey, 1 urban farm |

PUprdUr, UrFrc and Urprd are explained in Annex 5. *n* is the number of shops summed up. When *n* is a small number (qrB2.2, qrB2.3, qrC3, qrC4 and qrD1), the uncertainties were modelled using data of all shops of the same types. Uncertainty was not modelled for boiling centres (qrA1, qrB2) and retail shops with a bulk cooler purchasing from wholesaler (qrA3.1) and from peri-urban farms (qrC1) in order to keep separate because they were thought to be unique.

**Annex 4 Annual** average daily milk quantities passing through risk pathways

| Notation | Estimation (Litre) | Source |
| --- | --- | --- |
| qaA*i*  (Exception)  qaA1.2  qaA2.2  qaA3.3 | qrA*i* * mean of bootstrap of annual rain falls in Mbarara/ mean of bootstrap of rainy season rain falls in Mbarara  qaA1-qaA1.1  qaA2-qaA2.1-qaA2.3  qaA3-qaA3.1-qaA3.2 | Uganda Bureau of Statistics (2005) |
| qaB*i*  (Exception)  qaB1.1  qaB1.2  qaB2.1 | qrB*i** mean of bootstrap of annual rain falls in Namulonge/ mean of bootstrap of rainy season rain falls in Namulonge  If (qaA5+qaB1)>=qrB1.1*(qaA5+qaB1)/(qrA5+qrB1), qrB1.1*(qaA5+qaB1)/(qrA5+qrB1); if not, qaA5+qaB1  qaA5+qaB1-qaB1.1  qaB2-qaB2.2-qaB2.3 | Uganda Bureau of Statistics (2005) |
| qaC*i* | qrC*i** mean of bootstrap of annual rain falls in Namulonge/ mean of bootstrap of rainy season rain falls in Namulonge | Uganda Bureau of Statistics (2005) |
| qaD*i* | qrD*i** mean of bootstrap of annual rain falls in Namulonge/ mean of bootstrap of rainy season rain falls in Namulonge | Uganda Bureau of Statistics (2005) |

**Annex 5** Parameters of milk production

| Notation | Description | Estimation | Source |
| --- | --- | --- | --- |
| PUprdUr  LargePUprdUr  SmallPUprdUr  PUprdUrInf  LargePUInf  SmallPUInf  LFrc  SFrc  UrFrc  Urprd | Quantity produced by peri-urban farms and sold to urban areas  Quantity produced by 6 large PU herds selling to urban areas  Quantity produced by 36 small PU herds selling to urban areas  Quantity of infected milk produced by peri-urban farms and sold to urban areas  Quantity of infected milk produced by large PU herds and sold to urban areas  Quantity of infected milk produced by small PU herds and sold to urban areas  Reciprocal of large herds sample fraction  Reciprocal of small herds sample fraction  Reciprocal of urban farms sample fraction  Quantity produced by 54 urban farms | LargePUprdUr+SmallPUprdUr  ∑ bootstrap of quantity*LFrc*790/87  ∑ bootstrap of quantity*SFrc*790/87  LargePUInf+SmallPUInf  ∑ (bootstrap of quantity* RiskBinomial (1,3/6))  ∑ (bootstrap of quantity* RiskBinomial (1,4/117))  13/6  (625-13)/(177-6)  SFrc*790/87  ∑ bootstrap of quantity*UrFrc | Cattle survey  Cattle survey  Cattle survey  Cattle survey  Cattle survey  Cattle survey  Cattle survey  Cattle survey  Cattle survey  Cattle survey |

**Annex 6** Parameters of milk infection rate at origin of the pathways

| Notation | Description | Estimation | Source |
| --- | --- | --- | --- |
| irMb  irNL  irPU  irUr | Milk infection rate in Mbarara production areas:  - Mean 11.5% (90%CI: 3.9 – 20.6)  Milk infection rate in Nakasongola, Luweero production areas:  - Mean 25.0% (90%CI: 5.0 – 50.8)  Milk infection rate in peri-urban areas:  - Mean 25.3% (90%CI: 8.6 – 42.6)  Milk infection rate in urban areas:  - Mean 7.5% (90%CI: 1.0 – 16.6) | Manual construction of relative distribution  Manual construction of relative distribution  PUprdUrInf / PUprdUr  ∑ (bootstrap of quantity* RiskBinomial (1, 4/54))/ ∑ bootstrap of quantity | Milk IELISA  (8/57 positive)  Milk IELISA  (2/9 positive)  Cattle survey  Cattle survey |

**Annex 7**a Inactivation parameters

| Notation | Description | Estimation | Source |
| --- | --- | --- | --- |
| bMb  bA1  bA1.1  bA1.2  bA2  bA2.1  bA2.2  bA3  bA3.1  bA3.2  bA4  bNL  bB1  bB1.1 | Probability that milk produced in Mbarara is boiled  Probability that boiling centres in Nateete and Ndeeba boil milk  Probability that milk shops with a small refrigerator purchasing from boiling centre boil milk  Probability that vendors with a bicycle purchasing from boiling centre boil milk  Probability that retail bulk cooler milk shops purchasing from Mbarara boil milk  Probability that milk shops with a small refrigerator purchasing from retail bulk cooler shop boil milk  Probability that milk shops without a refrigerator purchasing from retail bulk cooler shop boil milk  Probability that wholesale milk shops in Nateete and Kitintale boil milk  Probability that retail bulk milk shops purchasing from wholesale shops boil milk  Probability that milk shops with a small refrigerator purchasing from wholesale shops boil milk  Probability that milk shops with a small refrigerator purchasing from Mbarara boil milk  Probability that milk produced in Nakasongola, Luweero is boiled  Probability that bulk cooler milk shops in Bwaise, Kawempe boil milk  Probability that milk shops with a small refrigerator purchasing from bulk cooler milk shops in Bwaise, Kawempe boil milk | 0  1  0  0  0  RiskBeta (4, 46)  RiskBeta (2, 3)  0  0  RiskBeta (4, 46)  RiskBeta (4, 46)  0  0  RiskBeta (4, 46) | Milk shop interviews  Interviews and observations  Milk shop interviews    Milk shop interviews  Milk shop interviews  Milk shop interviews, 3/48 shops with a small refrigerator purchasing fresh milk boiled  Milk shop interviews, 1/3 shops without a refrigerator purchasing fresh milk boiled  Milk shop interviews  Milk shop interviews  Milk shop interviews, as with bA2.1  Milk shop interviews, as with bA2.1  Milk shop interviews  Milk shop interviews  Milk shop interviews |

**Annex 7**b Inactivation parameters

| Notation | Description | Estimation | Source |
| --- | --- | --- | --- |
| bB2  bB2.1  bB2.2  bB2.3  bPU  bC1  bC2  bC3  bC4  bC5  bUr  bD1  bD2  bD3 | Probability that a boiling centre boils milk  Probability that vendors with a bicycle purchasing from a boiling centre boil milk  Probability that milk shops with a small refrigerator purchasing from a boiling centre boil milk  Probability that roadside milk vendors purchasing from a boiling centre boil milk  Probability that milk produced in PU is boiled  Probability that retail bulk cooler milk shops purchasing from PU areas boil milk  Probability that milk shops with a small refrigerator purchasing from PU areas boil milk  Probability that milk shops without a refrigerator purchasing from PU areas boil milk  Probability that roadside vendors purchasing from PU areas boil milk  Probability that vendors with a bicycle purchasing from peri-urban farms boil milk  Probability that milk produced in urban areas is boiled  Probability that vendors with a bicycle purchasing from urban farms boil milk  Probability that milk is boiled at the farm gate  Probability that milk is boiled before serving in large hotels | 1  0  0  0  0  0  RiskBeta (4, 46)  RiskBeta (2, 3)  0  0  0  0  0  1 | An interview and observation  Milk shop interviews  Milk shop interviews  Milk shop interviews  Cattle survey  Milk shop interviews  Milk shop interviews, as with bA2.1  Milk shop interviews, as with bA2.2  Interviews and observations  Cattle survey and interviews with vendors  Cattle survey  Cattle survey and interviews with vendors  Cattle survey  Cattle survey, hotels were not interviewed |
